# Latent class analysis of aggression in martial arts and combat sports: A cross-sectional study

**DOI:** 10.1371/journal.pone.0328799

**Published:** 2025-07-23

**Authors:** Cezary Kuśnierz, Marta Niewczas, Wojciech J. Cynarski, Grzegorz Bielec, Aleksandra M. Rogowska

**Affiliations:** 1 Faculty of Physical Education and Physiotherapy, Opole University of Technology, Opole, Poland; 2 Faculty of Physical Culture Sciences, University of Rzeszow, Rzeszow, Poland; 3 Institute of Psychology, University of Opole, Opole, Poland; Amazonas State University, BRAZIL

## Abstract

Previous research on aggression in martial arts and combat sports (MA&CS) has shown mixed results. Some studies showed that MA&CS training lowers aggression levels, while other studies found it increases aggression or has no effect. To explain better this inconsistency, this study used latent class analysis to identify distinct subgroups of MA&CS practitioners based on aggression levels and related demographic and sports factors. Previous research predominantly employed a variable-centered approach to investigate the relationships between variables and their impact on outcomes. This study adopts a person-centered approach to identify subgroups that exhibit similar patterns of aggression, thereby enhancing the understanding of individual differences through variable configurations. A cross-sectional study was conducted with 367 participants aged between 16 and 57 years old (*M* = 27.28, *SD* = 9.52), including 23% of women, and 76% MA&CS athletes in such disciplines as Brazilian jiu-jitsu (BJJ), karate Kyokushin (KK), mixed martial arts (MMA), and wrestling. Participants completed paper-and-pencil self-report psychological questionnaires, including the Buss-Perry Aggression Questionnaire and provided demographic information. Latent class analysis was performed using aggression scores, age, gender, education, economic status, MA&CS training experience, and discipline. Three latent classes were identified: MA&CS Experts (*n *= 182), MA&CS Newbies (*n* = 95), and Non-Athletes (*n* = 90). Among MA&CS Experts were more women [χ^2^(2) = 14.55, *p* < 0.001], older participants [H(2) = 236.42, *p* < 0.001], more experienced [H(2) = 8.31, *p* = 0.004], those with higher education [χ^2^(10) = 572.93, *p* < 0.001] and economic status [χ^2^(8) = 60.67, *p* < 0.001], and lower aggression scores [*F*(2, 161) = 10.443, *p* < 0.001], compared to MA&CS Newbies. MA&CS Newbies had higher physical aggression than Non-Athletes (*p* < 0.001). BJJ was overrepresented in the MA&CS Experts class, while KK and MMA were underrepresented [χ^2^(8) = 396.69, *p* < 0.001]. The MA&CS Newbies included athletes representing all four MA&CS disciplines in a similar proportion. The results highlight the role of long-term MA&CS training in potentially reducing aggression, particularly hostility, physical aggression, and verbal aggression. Demographic factors like age, gender, education, and economic status were also important in distinguishing the latent classes. The findings suggest aggression in MA&CS is a complex phenomenon influenced by multiple socio-cultural factors. Both the type of MA&CS and socio-demographic factors should be controlled by researchers and sports coaches if the goal of training is to reduce aggression in martial arts athletes.

## Introduction

### Characteristic of martial arts and combat sports

Martial arts are defined as systems that combine physical combat elements with philosophy, tradition, or other characteristics, thus distinguishing them from conventional physical exercises [1]. Although the term is predominantly applied to East Asian fighting systems, martial arts are universal cultural phenomena [2]. The foundation of acquiring proficiency in any martial art is systematic training, which typically imparts skills of self-regulation regarding aggressive impulses and self-defense techniques, enhances physical efficiency, and mitigates fear responses. In contemporary times, martial arts have evolved into combat sports, as exemplified by Olympic combat disciplines such as freestyle wrestling, Greco-Roman wrestling, boxing, fencing, judo, and taekwondo. Other martial arts, such as tai chi, have primarily developed as mind-body movement practices, de-emphasizing the pure combat aspect. In current academic literature, these disciplines are frequently referred to collectively as martial arts and combat sports (MA&CS) [3].

Combat sports (CS) require a high level of physical fitness, technical skill, and tactical acumen [4] CS encompasses a wide range of disciplines that involve physical confrontation between competitors. These sports are characterized by their unique techniques, rules, and physical demands, and they are often categorized based on the nature of the combat involved. Grappling sports, such as wrestling and Brazilian jiu-jitsu (BJJ), focus on techniques that involve holding and throwing [4–7]. Grappling sports require significant strength and endurance, as they often involve prolonged physical contact and control over the opponent. However, wrestling relies mainly on takedowns and pins [4–6], while BJJ focuses on ground fighting and submission holds and is known for its strategic approach to combat [4,7,8]. Striking sports, such as karate, emphasize hitting the opponent with various parts of the body, such as fists, elbows, knees, and feet [4]. Striking sports typically require speed, agility, and precision, with a focus on short and powerful movements [4,5,7]. Karate emphasizes speed and power and can be practiced both as a form of self-defense and a competitive sport [7,9]. Mixed martial arts (MMA) is a hybrid combat sport that combines techniques from both grappling and striking disciplines, like boxing, BJJ, and wrestling [7,8]. It requires a broad spectrum of physical and technical skills to succeed and is known for its versatility and complexity [4,6,7]. Furthermore, wrestling, BJJ, karate, and MMA can be considered as contact-based martial arts, which involve sparring and competitive bouts, increasing the risk of injuries, particularly to the head and extremities [10,11].

### Aggression in martial arts and combat sports

Human aggression is often categorized into two primary types: hostile and instrumental aggression [12–15]. Hostile aggression is impulsive and driven by the desire to harm, while instrumental aggression is premeditated and used as a means to achieve a goal. Instrumental aggression is goal-oriented and used strategically to achieve a specific objective, such as winning a match or defending oneself. It is often considered a necessary part of competitive sports, including martial arts, where the intent is to outperform an opponent rather than to cause harm for its own sake. Hostile aggression, in contrast, is characterized by an intent to harm or injure another person. This form of aggression can lead to negative consequences both for the aggressor and the victim, and it is generally discouraged in martial arts training.

Buss and Perry [16] distinguish four dimensions of aggression: physical aggression, verbal aggression, anger, and hostility. The Buss and Perry Aggression Questionnaire (BPAQ) was developed to assess these dimensions, which have been extensively studied and validated across different cultures and populations [16–19]. The four-factor structure has been confirmed through various factor analyses, demonstrating its robustness across different languages and cultural contexts, including Polish [20–22]. Gender differences were noted in validation studies, with higher scores of physical aggression among men than women [21].

Martial arts have long been debated for their impact on aggression, with some viewing them as a means to enhance self-control and reduce aggression and others as potentially promoting violent behavior. A meta-analysis of youth studies found that MA&CS can reduce externalizing behaviors such as aggression and anger, indicating a medium effect size [23]. Additionally, MA&CS participants generally exhibit less verbal aggression, hostility, and anger compared to non-participants [24]. However, the evidence is mixed, with some studies showing no significant relationship between martial arts practice and aggression levels [3,25]. Aggression can be linked to better performance in MA&CS and higher achievements during competitions [26,27]. Research suggests that the relationship between MA&CS and aggression is complex and multifaced. A recent systematic review found limited evidence for significant positive associations between MA&CS participation and externalizing emotion regulation, including verbal and physical aggression, hostility, and anger [28]. Moreover, experience in MA&CS training may moderate this relationship, with higher levels of competitive engagement and longer MA&CS practice being associated with lower aggression scores [28]. Numerous factors can contribute to aggression in MA&CS, including experience in MA&CS training, particular MA&CS discipline, age, gender, education level, and economic status.

### Factors influencing aggression among MA&CS practitioners

Cross-cultural studies performed among Hungarian, Latvian, Lithuanian, Polish, Romanian, Russian, Slovak, and Spanish martial arts athletes showed that country and specific culture may contribute in the aggressiveness of martial arts practitioners [29]. In particular, Spanish martial artists scored highest on the “Go-ahead” subscale, indicating a proactive approach to challenges, while Latvian practitioners scored highest on the “Foul play” subscale, indicating a higher tendency towards unethical behavior to achieve goals. Some research suggests that experience in MA&CS training is associated with lower levels of anger and aggression, especially in adults and young individuals with behavioral issues [3,30–34]. In particular, studies conducted in Poland, Romania and United States of America (USA) have shown that individuals participating in MA&CS training tend to exhibit lower levels of aggression than the non-training control groups [35–37]. Longitudinal research indicate that regular training in martial arts can help mitigate aggressive tendencies, particularly in younger practitioners [3]. However, the length of training plays a vital role in diminishing aggression, and incorporating internal techniques can further enhance this effect. Longer engagement in MA&CS generally correlates with decreased aggression, although this effect is more pronounced in traditional styles [23,24,38–41]. In particular, studies have shown that increased training in MA&CS correlates with lower levels of hostility and aggression among American and Polish athletes [24,40].

The discipline of MA&CS can influence aggression levels in Iranian, Hungarian and Polish studies [36,40,42,43]. For instance, after a year of training, Iranian participants in judo showed increased aggression, while those practicing karate did not experience significant changes in their aggression levels [42]. Adolescents from the USA who trained in MA (including aikido, iaido, judo, jiu-jitsu, karate, and kendo) showed lower levels of aggression on all scales of the BPAQ than their non-training peers [36]. However, CS competitors (including Muay Thai, boxing, fencing, and wrestling) scored significantly lower only in hostility than non-training individuals [36]. Similarly, MA practitioners demonstrated significantly lower levels of hostility and the general aggression index than CS athletes in a Polish study [40]. Furthermore, Polish male practitioners of MMA exhibit significantly higher levels of aggression compared to those practicing karate [43].

The age of martial arts practitioners significantly influences their aggression levels, with various studies highlighting different trends across age groups. Overall, younger practitioners tend to exhibit higher levels of aggression, which may decrease with maturity but can spike again in older age [41]. Studies also show that mid-adolescents (around 14 years old) exhibit the highest rates of aggression, likely due to hormonal changes and cognitive development challenges during puberty [41,42]. As adolescents mature into late adolescence (around 17 years old), their aggression levels typically decrease [42]. This pattern highlights the developmental factors influencing aggression during formative years. Moreover, younger individuals with behavioral issues may benefit more from martial arts training in terms of aggression reduction [23,40].

Gender also significantly influences aggression levels in martial arts, with males generally exhibiting higher levels of aggression than females across various studies [37,40,44]. Male MA&CS practitioners often score higher on such measures of aggression as physical aggression, verbal aggression, and hostility than their female counterparts [40,41]. However, aggression levels across various martial arts reveal distinct patterns influenced by training, style, and social factors [37,40,45–49]. For example, research comparing women practicing judo and BJJ found that women in judo exhibited higher levels of aggression, particularly in terms of hostility and anger. In contrast, women practicing BJJ showed a decrease in overall aggression levels over time, suggesting that the training environment and techniques may influence emotional outcomes differently [41,45]. Karli et al. [50] showed that while female athletes may exhibit increased physical aggression due to enhanced self-confidence from training, no significant differences were found between male and female combat sports athletes regarding their overall aggression levels. Female practitioners may experience different emotional outcomes than men, depending on the MA&CS styles, since karate promotes self-control, while BJJ may foster self-confidence that can lead to increased physical assertiveness. In addition, female combat athletes often demonstrate higher physical aggression levels than sedentary females but similar levels to male combat athletes [50]. Understanding these dynamics can help tailor martial arts training approaches to better suit individual needs across genders.

Socio-demographic factors, including education level and economic status, may influence perceptions of limitations, which in turn influence satisfaction and commitment to MA&CS practice, ultimately altering levels of aggression. Research suggests that education backgrounds can contribute to aggression among MA&CS athletes [37,40,44,51–53]. Polish studies have shown that university students and people with higher education participate in MA&CS internships significantly more often than those with primary and secondary education [52,53]. Several studies have directly explored how education influences aggression dimensions among athletes involved in martial arts and combat sports [37,40,44,51]. While higher education is correlated with lower aggression levels among some practitioners [37,40,44,51–53], the overall environment of martial arts training appears to foster reduced aggression through its emphasis on discipline and self-regulation [24]. For example, a recent study by Lafuente et al. [54] found no statistically significant differences in anger levels, anger expression, and anger control based on the level of formal education or years of experience of MA&CS practitioners. Further research is necessary to deepen the understanding of these relationships across various demographics and types of martial arts training.

Economic status may impact participation in MA primarily due to the costs associated with training and the availability of facilities. Biernat et al. [52,53] found that respondents with a good income level were significantly more likely to engage in MA&CS practices than those below and above a good earnings status. In addition, the study indicated that MA training sessions were mainly organized by sports clubs (36.4%), schools (19.2%), and individual organizers (18.8%). MA training sessions were usually held in sports centers (77.8%), and only for 25.3% of respondents, these trainings were free of charge. Economic conditions are a critical predictor of market demand for martial arts programs. Participants from varied economic backgrounds experience different levels of constraints, impacting their decision to continue or drop out [55]. Individuals with higher economic status are more likely to participate due to their ability to afford the associated costs [56].

### Justification for using a latent class analysis of aggression in this study

Aggression is inherent in sports competition, but its level varies depending on sports discipline and experience in MA&CS training, as suggested by studies [3,23,24,28,30–41].

In addition, research suggests that demographic variables, such as age, gender, education level, and economic status, can contribute to aggression among MA&CS practitioners. Previous studies used a variable-centered approach, which highlights the associations between variables and is commonly used to understand how different predictor variables contribute to an outcome [57]. The person-centered approach identifies subgroups of individuals who share similar attributes or patterns to understand individual differences by focusing on the holistic configuration of variables within subgroups [58,59]. However, recent advancements have led to the integration of person-centered and variable-centered approaches, allowing for a more comprehensive analysis using techniques such as latent class analysis [58,60]. This integration helps in addressing research questions that involve both individual differences and variable associations [60]. Latent class analysis can classify similar individuals into unique subpopulations based on very complex patterns of many variables [60]. The present study will use, for the first time, the latent class analysis to examine whether individual differences in aggression, as well as socio-demographic and sport-related variables, can explain the inconsistency in aggression levels among MA&CS practitioners. We assume that there are distinct subpopulations within the MA&CS athletes that share specific traits based on inherited and contextual characteristics of behavior and emotion, which manifest in the selection of a given MA&CS discipline to train, and some socio-demographic variables, including age, gender, education level, and self-reported economic status. The study has an explorative character; therefore, no specific hypotheses have been formulated. The effectiveness of MA&CS training largely depends on the type of martial art, the existing traits of the participants, and the teaching approach of the instructors. We believe that the results of this study can be helpful in managing aggression among individuals practicing MA&CS and improving teaching particular MA&CS among coaches.

## Materials and methods

### Study design and procedure

Cross-sectional studies were performed in Poland between 15 February and 30 September 2024. The research protocol was approved by the Research Ethics Committee at the Opole University of Technology (Decision No. 2/2022, 9 July, 2022). Written informed consent was obtained from all participants before starting the study. With the consent of the coaches, MACS athletes were given paper and pencil questionnaires distributed after the training session in the sports clubs. The study covered MA&CS athletes from the Opole and Podkarpackie provinces who attended training camps and seminars devoted to improving combat techniques and exchanging experiences in the field of sports training theory. These were clubs: Forca Opole – (BJJ/MMA/Wrestling), Forca Nysa – (BJJ), Heel Hoo Namysłow (BJJ/ Wresling), Next Level – (BJJ/MMA), Orzeł Namysłów – (Wresling), Klub Karate Kyokushin Sandomierz, Boxing/MMA Club “Wisłok” Rzeszów. Their diversity and separate combat rules dictated the selection of MA&CS disciplines for the present research. MA&CS discipline is an essential factor influencing the training process: technical, strength, and endurance preparation, as well as shaping mental resilience in players fighting in contact. We assume that these various aspects of sports training will influence sports achievements as well as differences in managing aggression. The inclusion criteria for the martial arts athletes groups were: at least 16 years old, at least a half year of practice of martial arts in a sports club in one of the following MA&CS fight style: Brazilian Jiu-Jitsu (BJJ), Karate Kyokushin, wrestling, or mixed martial arts (MMA). In addition, a control group included people not practicing MA&CS. A group of informatic technologies students participated in the study at the Opole University of Technology. The inclusion criteria for the control group were as follows: age of at least 16 years and no MA&CS practice.

The sample size was determined *a priori* using G*Power software [61]. Considering moderate effect size for one-way ANOVA (ƒ = 0.25), five groups for comparison, the minimal value of *p* = 0.05, and power 0.95, the expected sample size was *N* = 305. For Pearson’s χ^2^ test of independence, when assuming moderate effect size (ω = 0.30), *df *= 5, the minimal value of *p* = 0.05, and power 0.95, the expected sample size was *N* = 220. It isn’t easy to assess the statistical power of LCA, but results from simulation studies indicate a sample size between 300 and 1,000 individuals as appropriate for desirable power [62,63].

The invitation to the study accepted 400 people. However, four people refused to participate, and 29 people were rejected because they were underage or practiced a sport or a fighting style that did not meet the inclusion criteria. The final sample included 367 subjects, which is more than sufficient for adequate power.

### Demographic characteristics of the sample

The total sample consisted of 367 participants, including 277 MA&CS athletes (75.47% of the total sample) aged between 16 and 57 years old (*M* = 27.28, *SD* = 9.52). The majority of the respondents were men with higher education levels and good self-reported economic status (Table 1). Among MA&CS competitors, the majority practiced Brazilian Jiu-Jitsu (*n* = 107; 29.16% of the total sample), followed by wrestling (*n* = 78, 21.25%), MMA (*n* = 50, 13.62%), and karate (*n* = 42, 11.44%). The sports experience of MA&CS practitioners ranged between half and 41 years, and on average, it was 8 years (*M* = 7.65, *SD* = 5.68). Most athletes participated in championships at the national level of competitions. The sample of non-athletes consisted of university students of informatic technologies (*n* = 90, 24.52%), most of them under Bachelor’s degree (*n* = 66) or Master’s degree study levels (*n* = 25).

**Table 1:** Sample characteristic (*N* = 367).

Variable	Categories	Range/ coding	MA&CS		Non-athletes	
			*M/n*	*SD*/%	*M/n*	*SD*/%
Age		16-57	28.81	10.12	22.60	5.07
Sex	Women	0	71	25.632	15	16.67
	Men	1	206	74.368	75	83.33
Education	Primary	0	54	19.50	0	0
	Vocational	1	16	5.78	0	0
	Secondary	2	99	35.74	0	0
	Under Bachelor’s	3	1	0.36	65	72.22
	Under Master’s	4	0	0.00	25	27.78
	Master’s or higher	5	107	38.63	0	0
Subjective assessment of economic status	Disappointing	0	11	3.97	5	5.56
	Average	1	26	9.39	26	28.89
	Good	2	136	49.10	48	53.33
	Very good	3	82	29.60	9	10.00
	Excellent	4	22	7.94	2	2.22
Type of martial arts	Non-athlete	0	90	24.52		
	Brazilian Jiu-Jitsu	1	107	29.16		
	Karate Kyokushin	2	42	11.44		
	Wrestling	3	78	21.25		
	MMA	4	50	13.62		
Championship range	None	0	90	24.52		
	Regional	1	87	23.71		
	National	2	146	39.78		
	European	3	34	9.26		
	World	4	10	2.73		
Number of minutes PA per week		0-8400	1407.55	1629.88		
Sports experience (years)		0.5-41	7.65	5.68		

*Note*. MA&CS = martial arts and combat sports, MMA = mixed martial arts, PA = physical activity, *M* = mean, *n* = number of participants, *SD* = standard deviation.

### Measures

The Buss and Perry Aggression Questionnaire (BPAQ) is a 29-item self-report measure designed to assess various aspects of aggression [16]. The BPAQ consists of four components: physical aggression ([PA]; general tendency to engage in physical acts of aggression, e.g., “If I have to resort to violence to protect my rights, I will”), verbal aggression ([VA], a propensity to engage in verbal arguments and confrontations, e.g., “I tell my friends openly when I disagree with them”), anger ([A], the emotional aspect of aggression, including quick temper and frustration, e.g., “Some of my friends think I am a hothead”), and hostility ([H], feelings of ill will and suspicion towards others, e.g., “When people are especially nice to me, I wonder what they want”). Participants use a 5-point Likert scale (1 = *Extremely Uncharacteristic* to 5 = *Extremely Characteristic*) to indicate how well a statement describes them. Higher scores (ranging between 29 and 149) indicate greater endorsement of aggressive statements and indicate a greater propensity for aggressive thoughts, feelings, and behaviors. Adequate to strong internal consistency has been observed for the total scale as well as all four subscales in previous studies, including Polish validation [20], which was used for the present study. In the current study, reliability (McDonald’s ω) was 0.88 for the total BPAQ score and 0.76, 0.58, 76, and 79 for PA, VA, A, and H subscales, respectively.

The socio-demographic questions concerned age (number of completed years), gender (Men, Women, Other), education level (Primary, Vocational, Secondary, Under Bachelor’s degree studies, Under Master’s degree studies, Master’s degree or higher), and self-reported subjective assessment of economic status (Disappointing, Average, Good, Very good, Excellent). Among MA&CS athletes, the questions were related to sports discipline (BJJ, Karate, Wrestling, MMA), the highest championship range (None, Regional, National, European, World), the number of minutes of physical activity per typical week, and MA&CS sports experience (years).

### Statistical analysis

As a preliminary analysis, we examined parametric properties of the BPAQ scales, including mean (*M*), standard deviation (*SD*), skewness, and kurtosis. Since the sample size was large (*N* = 367), and skewness (from 0.05 to 0.56) and kurtosis (from 0.69 to 0.09) ranged between +/- 1, we assumed that parametric test could be used in the next steps of statistical analyses. There were no missing data in the study. Latent class analysis (LCA) was performed based on the glca R package of the JAMOVI software, ver. 2.3.28. The LCA was conducted for four categorical manifest variables (gender, education level, economic status, and MA&CS discipline) and six continuous variables considered as covariates (age, experience in MA&CS training, and four subscales of aggression: PA, VA, A, and H). We started analysis from 4 latent classes, and the final LCA was performed for three classes, which presented the best parameters compared to other options. As a sensitivity analysis, Pearson’s χ^2^ test of independence was used for all categorical variables to compare three latent profiles across gender, education level, subjective assessment of economic status, the discipline of MA&CS, and range of championships, in which martial arts athletes were engaged at the highest level in their life. The Kruskal-Wallis H test was conducted to examine differences in age, experience in MA&CS practice, and number of hours spent on physical activity during a typical week between three latent profiles. Finally, one-way ANOVA was performed to compare aggression scales across three latent classes. The Games-Howell posthoc test was used for the comparison of aggression levels between particular samples, and partial eta-square (η²_*p*_) was used to assess effect size.

## Results

### Determining the number of latent classes

**Fig 1:**
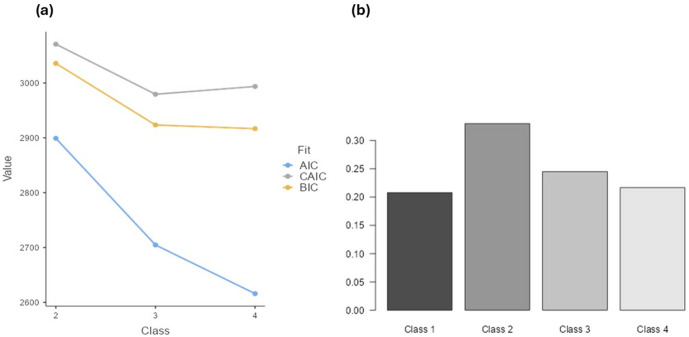
Elbow plot (a) and class prevalence (b) for five latent classes solution, determining the number of profiles.

The LCA was started from a 4-class solution to determine an appropriate number of classes. The results are presented in Table 2. The elbow plot indicates that three classes fit the data well (Fig 1), taking into account that the lowest value was for CAIC for a three-class solution. Therefore, a three-class solution was assumed appropriate and will be considered in the next steps of statistical analyses. The prevalence of participants in each group representing latent class (LC) is 90, 182, and 95 for LC_1_, LC_2_, and LC_3_, respectively. The characteristics of each profile, in terms of demographics and aggression levels, will be examined in the following paragraphs.

**Table 2:** Absolute model fit for five latent classes solution.

Class	Parameter	Log-likelihood	AIC	CAIC	BIC	Entropy	*df*	G²	*p*
2	35	−1414.55	2899.11	3070.80	3035.80	1.00	331.00	2829.11	0.32
3	56	−1296.40	2704.79	2979.49	2923.49	0.96	310.00	2592.79	NaN
4	77	−1231.00	2615.99	2993.71	2916.71	0.93	289.00	2461.99	NaN

*Note.* AIC = Akaike Information Criterion, CAIC = , BIC = Bayesian Information Criterion, *df* = degrees of freedom, G2 = likelihood ratio statistics, *p* = level of statistical significance.

### Demographic profile of the three latent classes

Validation of the LCA model was performed by comparison of three latent profiles in terms of such demographic characteristics of participants, such as gender, education level, subjective assessment of economic status, type of martial arts, and range of championships, in MA&CS athletes were engaged at the highest level in their life. All these group differences were significant. Pearson’s χ^2^ test of independence was used for all these categorial variables (Table 3). Statistically, more women are included in the LC3 than in LC1 and LC2. Significant differences were found in levels of education. Self-reported disappointing and average economic status was presented statistically more frequently in samples LC1 and LC2, while very good and excellent in group LC3. The LC2 sample contains individuals representing all MA&CS disciplines in a similar proportion. In contrast, proportionally, there were many more BJJ athletes, while fewer karate and MMA practitioners were included in LC3. Significantly more MA&CS competitors with a higher European range of championships were presented more frequently in the LC3 group than in the LC2 sample. In contrast, significantly more athletes competing at the regional range were included in the LC2 group than in the LC3 sample (Table 3).

**Table 3:** Pearson’s χ^2^ test of independence, comparing demographic characteristics of three latent profiles (*N* = 367).

Variable	LCA Membership						Total		χ^2^	*df*	*p*	CC
	LC1		LC2		LC3		(*n* = 367, 100%)					
	(*n* = 90, 24.52%)		(*n* = 95, 25.89%)		(*n* = 182, 49.59%)							
**Sex (total)**									14.55	2	< 0.001	0.20
Women	15	4.1 %	13	3.5 %	58	15.8 %	86	23.4 %				
Men	75	20.4 %	82	22.3 %	124	33.8 %	281	76.6 %				
**Education (total)**									572.93	10	< 0.001	0.78
Primary	0	0.0 %	54	14.7 %	0	0.0 %	54	14.7 %				
Vocational	0	0.0 %	5	1.4 %	11	3.0 %	16	4.4 %				
Secondary	0	0.0 %	35	9.5 %	64	17.4 %	99	27.0 %				
Under Bachelor’s	65	17.7 %	1	0.3 %	0	0.0 %	66	18.0 %				
Under Master’s	25	6.8 %	0	0.0 %	0	0.0 %	25	6.8 %				
Master’s degree or higher	0	0.0 %	0	0.0 %	107	29.2 %	107	29.2 %				
**Economic status (total)**									60.67	8	< 0.001	0.38
Disappointing	5	1.4 %	9	2.5 %	2	0.5 %	16	4.4 %				
Average	26	7.1 %	17	4.6 %	9	2.5 %	52	14.2 %				
Good	48	13.1 %	47	12.8 %	89	24.3 %	184	50.1 %				
Very good	9	2.5 %	19	5.2 %	63	17.2 %	91	24.8 %				
Excellent	2	0.5 %	3	0.8 %	19	5.2 %	24	6.5 %				
**Type of martial arts**									396.69	8	< 0.001	0.72
None	90	24.5 %	0	0.0 %	0	0.0 %	90	24.5 %				
Brazilian Jiu-Jitsu	0	0.0 %	22	6.0 %	85	23.2 %	107	29.2 %				
Karate Kyokushin	0	0.0 %	23	6.3 %	19	5.2 %	42	11.4 %				
Wrestling	0	0.0 %	25	6.8 %	53	14.4 %	78	21.3 %				
Mixed martial arts	0	0.0 %	25	6.8 %	25	6.8 %	50	13.6 %				
**Championship range**									378.38	8	< 0.001	0.71
None	90	24.5 %	0	0.0 %	0	0.0 %	90	24.5 %				
Regional	0	0.0 %	37	10.1 %	50	13.6 %	87	23.7 %				
National	0	0.0 %	49	13.4 %	97	26.4 %	146	39.8 %				
European	0	0.0 %	5	1.4 %	29	7.9 %	34	9.3 %				
World	0	0.0 %	4	1.1 %	6	1.6 %	10	2.7 %				

*Note*. LC = latent class, LCA = latent class analysis, *n* = number of participants, χ^2^ = Pearson’s Chi-square test of independence, *df* = degrees of freedom, *p* = level of statistical significance, CC = contingency coefficient as an effect size for Pearson’s Chi-square test of independence.

In addition, the Kruskal-Wallis H test was conducted for age, sports experience (in years of practice in martial arts), and sport engagement (measured by number of hours spent on physical activity during a typical week) across three latent profiles. The Kruskal-Wallis H is a nonparametric alternative for the analysis of variance (ANOVA). Significantly lower age was represented by LC2 group (*n* = 95, *M* = 18.74, *SD* = 3.17), compared to LC1 (*n* = 90, *M* = 22.60, *SD* = 5.07), and also LC3 samples (*n* = 182, *M* = 34.06, *SD* = 8.36). All these differences were statistically significant and showed a large effect size, H(2) = 236.42, *p* < 0.001. The sample LC2 presented significantly shorter experience in MA&CS (*n* = 95, *M* = 6.31, *SD* = 4.31) than those of LC3 group (*n* = 182, *M* = 8.41, *SD* = 6.16), H(2) = 8.31, *p* = 0.004. However, MA&CS athletes of LC2 (*n* = 95, *M* = 2086.42, *SD* = 1763.76) did not differ significantly from those of LC3 (*n* = 182, *M* = 1749.23, *SD* = 1552.98) in terms of number of minutes per week spend on sport training. In contrast, both athlete groups LC2 and LC3 differ significantly from student sample LC1 (which was not exercised at all), H(2) = 207.95, *p* < 0.001.

### Comparison of three latent classes in terms of aggression level

**Fig 2:**
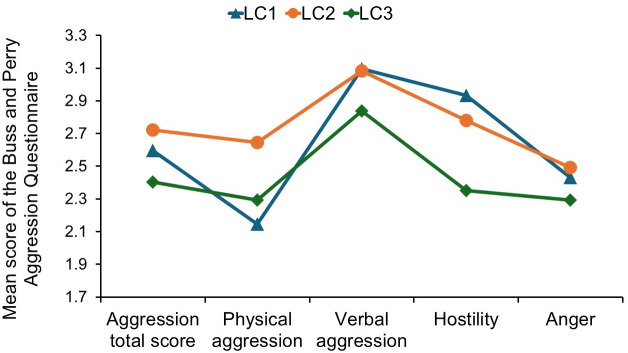
Mean scores in the total level of aggression and subscales of the Buss and Perry Aggression Questionnaire (physical aggression, verbal aggression, anger, and hostility) across three profiles representing latent class analysis.

One-way analysis of variance showed that all aggression scales, except the anger subscale, differed significantly across the three latent classes (Table 4 and Fig 2). The Games-Howell posthoc test showed that the LC3 sample scored significantly lower in the total aggression score than both groups LC1 (*p* = 0.021) and LC2 (*p* < 0.001), but no differences were found between LC1 and LC2. Significantly higher physical aggression was shown in the LC2 sample than in LC3 (*p* < 0.001) and LC1 (*p* < 0.001), while these two groups, LC1 and LC3, did not differ from each other. Taking into account verbal aggression, LC3 scored significantly lower than LC1 (*p* = 0.023) and LC2 (*p* = 0.011). Samples LC2 and LC1 showed almost identical scores in verbal aggression. Hostility level was significantly lower in the LC3 sample, compared to both LC1 (*p* < 0.001) and LC2 (*p* < 0.001), and these two groups, LC1 and LC2, did not differ from each other. Anger was unrelated to LCA profiles.

**Table 4:** The one-way ANOVA to compare aggression across three latent classes (LC) (*N* = 367).

Variable	LC1 (*n* = 90)		LC2 (*n* = 95)		LC3 (*n* = 182)		*F*(2, 161)	*p*	η²_p_
	*M*	*SD*	*M*	*SD*	*M*	*SD*			
Aggression – total score	75.27	15.71	78.92	16.13	69.65	17.15	10.443	< 0.001	0.055
Physical Aggression	19.32	5.57	23.82	5.58	20.64	5.86	16.377	< 0.001	0.079
Verbal Aggression	15.48	3.90	15.42	3.27	14.19	3.45	5.922	0.003	0.031
Hostility	23.46	6.58	22.22	5.93	18.79	6.64	18.304	< 0.001	0.094
Anger	17.01	5.50	17.45	5.28	16.04	5.63	2.334	0.100	0.012

*Note*. LC = latent class, *n* = number of participants, *M* = mean, *SD* = standard deviation, *F* = Fisher’s test, *p* = level of statistical significance, η²_p_ = partial eta-square.

## Discussion

The study used LCA to identify qualitatively different subgroups within the MA&CS sample. Based on previous research, we assumed that MA&CS practitioners could share some socio-demographic (age, gender, education level, and economic status) and sport-related characteristics (i.e., MA&CS training experience and distinct disciplines among BJJ, karate, wrestling, and MMA), related to aggression levels. The LCA found three distinct unobserved (latent) groups, classified as LC1, LC2, and LC3. By statistically identifying latent classes, the study aimed to understand the diversity within MA&CS athletes better, as compared to non-athletes, based on many easy-to-identify demographic characteristics. The LCA revealed three subtypes related to aggression levels. A person-centered training approach could then address the unique traits of individuals in each subtype.

To summarize the results of this study, the LC3 sample consisted mainly of older athletes with relatively extensive experience in sports training, a higher range of sports competitions, and representing to a greater extent BJJ and to a lesser extent karate and MMA, compared to the LC2 sample. The LC3 group also declared higher education levels and satisfaction with their economic status, compared to LC1 and LC2. The LC3 group showed the lowest level of general aggression as well as significantly lower levels of physical aggression, verbal aggression, and hostility than the other groups. Notably, relatively more women were included in the LC3 than in the LC2 and LC1 samples. We can call this group as “MA&CS Experts”. The LC2 group consisted mainly of young athletes at the beginning of their MA&CS sports career, as evidenced by the relatively low level of sports competitions and short sports experience. These individuals were evenly distributed across groups representing individual MA&CS disciplines, with no one discipline dominating. The LC2 group represented the lowest level of education and generally low satisfaction with their economic status, as well as significantly higher levels of general, physical, and verbal aggression, as well as hostility, compared to the LC3 athlete group. Moreover, the LC2 athlete group did not differ significantly from the non-athlete control group in terms of the overall level of aggression, as well as on the verbal aggression and hostility scales. Even in terms of physical aggression, the LC2 group showed higher levels compared to the control group. We propose to call this LC2 sample the group of “MA&CS Newbies.” The sample LC1 consisted of IT students and was called “MA&CS Non-Athletes”. This group differed mainly from the well-trained LC3 in terms of aggression level.

### Latent profiles across socio-demographic and sport-related characteristics

The present research seems to be in line with previous studies, which systematically demonstrated that younger age is related to higher aggression [23,28,40–42]. Research indicates that physical aggression levels tend to decrease with age until approximately 55 years, after which they may increase again [41]. The highest levels of anger are observed in younger practitioners (ages 10–15) and older adults (ages 56–79). It may suggest a curvilinear relationship where aggression is more pronounced at the extremes of age. However, aggressive behaviors established in childhood often persist into adulthood. Individuals who exhibited higher levels of aggression at a young age tend to maintain those patterns later in life, suggesting that early interventions through martial arts training could be beneficial for managing aggression [41].

We found relatively more women among the “MA&CS Experts” than “MA&CS Newbies” and “MA&CS Non-Athletes”. It is important to note that overall, fewer women participated in the study than men. However, the gender imbalance in this study reflects the lower participation of women in both MA&CS training and within the IT industry. This result supports some previous research performed among MA&CS competitors but is inconsistent with other studies. Overall, men have been found to score higher on measures of physical aggression, verbal aggression, and hostility than their female counterparts [37,44]. Combat sports have traditionally been viewed as male-dominated arenas, but recent research highlights the evolving gender dynamics within these sports. Gender differences have been noted, with female martial artists generally exhibiting lower aggression levels compared to males. It includes lower levels of physical and verbal aggression, hostility, and overall aggression index [40]. Also, the study performed among Hungarian adolescents (aged between 14 and 18) showed that boys scored significantly higher than girls in total aggression and physical aggression while lower in anger and verbal aggression compared to girls [36]. Interestingly, in the sample of MA&CS practitioners, only physical aggression was significantly lower among girls than in the boys group. In contrast, neither the total score of BPAQ nor subscales of anger, hostility, and verbal aggression differed across genders [36].

While men generally exhibit higher aggression levels than women across various martial arts, participation in these activities can lead to significant reductions in overall aggressiveness for both genders. Women participating in combat sports tend to show increased physical aggression compared to non-practitioners but do not exhibit higher overall aggression than male counterparts [41,50]. Furthermore, female combat athletes often display higher physical aggression levels compared to sedentary females, indicating that training can enhance self-confidence and assertiveness. However, this increase is typically not accompanied by a rise in overall aggression compared to male athletes [41,50]. A study involving various combat sports (including Wushu, Taekwondo, Judo, and Boxing) indicated no significant difference in aggression levels between male and female athletes. Both genders displayed similar mean scores for aggression, suggesting that participation in combat sports may level the playing field regarding aggressive behavior [46,47].

The MA&CS discipline may also influence differences in aggression levels between women and men. For example, a study indicated that female karate practitioners reported higher levels of anger and hostility compared to male practitioners [37]. Specifically, females scored significantly higher on measures of anger (*M* = 16.6) and hostility (*M* = 20.8) than males (*M* = 11.2 and *M* = 13.4 respectively) [37]. Despite this, both genders experienced lower overall aggression levels due to the self-regulatory nature of karate training. However, BJJ training appeared to result in lower aggression levels among women compared to judo training [45]. In particular, women practicing judo report higher levels of hostility and anger than those practicing BJJ, highlighting the impact of training style on emotional regulation. In many cases, martial arts training can reduce gender disparities in aggression levels, allowing both men and women to develop similar emotional responses through discipline and self-control. The specific martial art practiced influences the degree and type of aggression exhibited, with styles like BJJ promoting lower aggression levels among female practitioners compared to judo [45].

Cultural factors also play a crucial role in shaping how aggression is expressed within martial arts contexts. Societal norms and cultural expectations play a significant role in shaping aggression behaviors and significantly influence how aggression is expressed among genders. Women are often socialized to suppress overt aggression, leading to different manifestations of aggressive behavior compared to men [40,41]. Women typically receive more negative feedback regarding physical aggression during development, leading to a cultural tendency for women to express aggression less overtly compared to men. This cultural backdrop influences how aggression manifests in martial arts settings. Biological factors such as testosterone levels are often cited as contributing to higher aggression in men. However, socialization and cultural acceptance also play critical roles in how both genders express and manage aggressive impulses [44].

The study indicates that educational background can be necessary for profiling MA&CS practitioners and non-athletes. Lower education levels were generally found in the LC2 than in the LC3 athletes groups. Furthermore, the LC3 group with general higher education levels was twice as large as LC2 in this study. Research conducted among 6,547 residents of the capital of Poland, Warsaw, showed that university students (4.7%) practice martial arts (MA) significantly more often than people with primary/vocational education (1.1%) as well as those with higher (1.8%) and secondary education (2.5%) [53]. Also, a nationwide survey in Poland revealed that MA training was more likely among university students and people with higher education status, as compared to those with primary and secondary education [52].

The relationship between education and aggression among MA&CS practitioners was also examined. Research has shown a statistically significant relationship between higher education levels and decreased aggression among judo athletes, indicating that education may play a role in moderating aggressive behaviors [40]. This correlation was not observed in the control group, highlighting the importance of context in understanding these dynamics. In a study performed among Hungarian adolescents, students of vocational school had the highest trait of aggression compared to those from technical and high school students [44]. Education can shape individuals’ perceptions of aggression through social learning. Practitioners with higher educational backgrounds may be more likely to adopt non-aggressive behaviors modeled by instructors or peers, further reducing aggressive tendencies [37].

Education level influences aggression in martial arts practitioners in various ways, with research indicating that higher levels of education are often associated with lower aggression levels. Research indicates that the number of years spent practicing MA correlates positively with self-control and negatively with aggression. Practitioners with more extensive training tend to demonstrate lower aggression levels, suggesting that both education and training experience contribute to emotional regulation [40,64]. However, Lafuente et al. [65] examining anger expression and emotional control, found no significant differences in anger or aggression levels based on the level of formal education among MA&CS practitioners. It may suggest that education level did not significantly affect these traits among male practitioners. While education may influence aggression indirectly through self-control, it may not be a direct determinant. The educational background of martial artists is an area noted for further exploration. Some studies advocate for additional research to assess how different educational experiences might affect aggression levels across diverse populations practicing martial arts [40].

Also, self-reported economic status varies across latent profiles. Members of the LC3 profile were generally given higher satisfaction with their economic levels than the LC2 and the LC1 samples. While direct research on the relationship between economic status and aggression in martial arts practitioners is limited, several factors suggest that economic background can influence access to training, socialization processes, mental health outcomes, and community support systems, all of which can impact aggression levels. Individuals from lower socioeconomic backgrounds may face challenges that could hinder the positive effects of martial arts training on aggression reduction. Understanding these dynamics is essential for developing inclusive programs that promote emotional regulation and self-control across diverse populations.

Economic status can also affect access to martial arts training. Higher socioeconomic groups may benefit from more supportive training communities that foster positive emotional development, while lower socioeconomic groups might face more challenges, such as exposure to stressors that can increase aggressive behaviors. Individuals from lower socioeconomic backgrounds may have limited opportunities to participate in martial arts due to costs associated with classes, equipment, and facilities [52]. This lack of access could lead to fewer opportunities for learning self-control and emotional regulation through martial arts, potentially resulting in higher aggression levels compared to those with more resources. Martial arts are often perceived as niche sports with high costs, which can discourage participation. It is especially evident in Poland, where the lack of affordable facilities is a barrier to continued participation after years of study. [52]. The availability of affordable martial arts facilities near one’s residence or workplace is crucial. The lack of such facilities can disproportionately affect those with lower economic status, limiting their access to martial arts training [52].

Participants from economically disadvantaged backgrounds may experience different socialization processes influencing their aggression levels. Exposure to violence or aggression in their environment may lead to similar behaviors. In contrast, those from higher socioeconomic backgrounds may access supportive environments promoting non-aggressive behaviors through structured activities like martial arts. Additionally, individuals from lower economic backgrounds may face stressors (e.g., financial instability) counteracting the positive effects of martial arts training on aggression. Economic status can influence the martial arts community individuals are part of. Those with higher economic status may access more organized, supportive training environments fostering positive social interactions and reducing aggressive tendencies. Conversely, practitioners from lower socioeconomic backgrounds might encounter less structured environments not emphasizing emotional regulation or conflict resolution. The type of martial art practiced may also interact with economic status in influencing aggression levels. Traditional martial arts often emphasize discipline and respect, mitigating aggressive behaviors regardless of economic background. In contrast, more competitive or aggressive styles (like MMA) might attract individuals exhibiting higher aggression levels or from more aggressive environments.

Martial arts disciplines vary in popularity among economic groups based on accessibility, cultural significance, and costs. Combat sports like MMA and BJJ may have a higher concentration of middle to upper-class practitioners due to associated costs and facilities. Karate and taekwondo schools are prevalent in many communities. Their low equipment requirements make them more affordable for families with limited resources. BJJ attracts practitioners from diverse backgrounds but tends to draw more middle to upper-class individuals due to training and competition costs. BJJ schools often charge higher tuition than traditional dojos, limiting access for lower-income individuals. MMA has gained popularity across demographics, including lower socioeconomic backgrounds. However, costs for gym memberships and specialized equipment can be a barrier. High-income individuals may be drawn to MMA for its competitive nature and opportunity to engage in a multifaceted combat sport combining various martial arts techniques.

In terms of the MA&CS discipline, the study found that the LC3 profile included relatively more BJJ athletes but fewer karate and MMA athletes than the LC2 sample. Wrestling did not differentiate athletes in terms of sample size in the LC3 and LC2 profiles. Notably, all non-athlete IT university students were included in one LC1 group. In addition, significant differences were found between these profiles in terms of total aggression level and subscales of physical aggression, verbal aggression, and hostility. Only anger did not differentiate the latent profiles. This study’s results seem to confirm the key role of MA&CS in altering aggression. The remaining results, concerning sport-related variables, seem to be related to the vital role of training in reducing aggression levels on the hostility dimension. However, physical aggression, which is the essential means to success in combat sports, remained at the highest level in MA&CS athletes.

A recent systematic review [28] found mixed results for associations between type of MA&CS and various aspects of aggression. For example, the evidence remains inconclusive regarding whether karate practitioners demonstrate different levels of aggression compared to non-athletes. However, participation in karate is consistently associated with lower aggression levels than those observed in kickboxing, judo, and wrestling [28]. Furthermore, hostility does not differentiate between karate and BJJ athletes. However, beginners in karate and BJJ showed higher rates of hostility compared to intermediate and advanced level practitioners, as well as to non-practicing individuals [28].

However, a meta-analysis by Vertonghen and Theeboom [33] reviewed 350 studies and found that more extended martial arts training is generally associated with lower levels of aggression. This comprehensive analysis supports the idea that sustained engagement in martial arts can lead to significant reductions in aggressive behaviors over time. While the consistency and robustness of the evidence are limited, recent systematic reviews suggest that the length of MA&CS training is associated with reduced aggression levels [28]. Research conducted by Zivin et al. [34] indicated that adolescents participating in traditional martial arts programs experienced decreases in violent behavior and aggression over a school year. It may suggest that traditional martial arts, which often emphasize respect and self-discipline, can be effective in mitigating aggressive tendencies among youth. The reduction in aggression over time may be attributed to the development of self-regulatory skills and emotional management techniques learned through martial arts practice. As practitioners gain experience, they often become better equipped to handle conflicts without resorting to aggressive behavior.

For instance, a previous study indicated that MA apprentices exhibited significantly lower hostility and general aggression compared to CS athletes, suggesting that sustained engagement in martial arts fosters emotional regulation and reduces aggressive behaviors over time [40]. Research highlights that longer training durations, such as 10 months or more, are particularly effective in reducing aggression among participants. This effect is attributed to the structured environment of martial arts training, which emphasizes discipline, respect, and self-control [23].

Many studies have shown that increased duration of martial arts training correlates with lower levels of aggression [23,28,40]. Decreased aggression associated with long-term training is observed across various MA&CS disciplines, suggesting that sustained practice may foster emotional self-control and reduce hostility [30,31,39,66]. Furthermore, intensity of training does not significantly affect aggression levels, but the inclusion of internal techniques (e.g., breathing exercises) can reduce state hostility and aggressive behavior [38,67]. Also, incorporating philosophical teachings alongside physical skills in martial arts can significantly reduce both reactive and proactive aggression, especially in children and adolescents [68].

Aggression is closely related to mental health through mechanisms involving executive dysfunction and impulsivity [69]. Executive functions increase with age and brain maturation [41,42]. Younger MA&CS practitioners show higher aggression, which decreases with maturity but can increase in older age [41,69]. Deficits in executive functions, particularly in the prefrontal cortex, are linked to aggressive behavior [70]. Executive dysfunction, specifically in working memory, has been identified as a predictor of reactive aggression, indicating that impairments in these cognitive processes can lead to increased aggression in response to provocation [71]. Furthermore, increased risk-taking in decision-making is positively correlated with higher levels of aggression, particularly reactive aggression, suggesting that impulsivity plays a significant role in aggressive behaviors [72,73]. The mental state of an athlete, particularly in terms of anxiety, motivation, and emotional regulation, can significantly influence their performance [28,69,74,75]. In particular, athletes tend to experience heightened tension, anger, anxiety, and nervousness when they are losing [74]. Conversely, when they perform well, these emotions diminish, and their motivation increases. Therefore, it is crucial to monitor and train these psychological factors [74].

Previous research has shown that contact sports increase aggression levels, while non-contact sports improve self-control [76,77]. Overall, MA&CS can enhance conflict resolution skills and emotional control [25]. While some studies show a reduction in aggression, others find the opposite or insignificant relationship between martial arts practice and aggression levels, highlighting the need for more research to understand the conditions under which martial arts can be beneficial [3,33]. The effects on aggression can vary significantly based on the type of martial arts practiced and the context in which training occurs, such as the presence of philosophical teachings or therapeutic elements [23,28,31,67,78]. For example, it was found that modern martial arts, such as MMA, may not be suitable for at-risk youth as they can lead to increased aggression, especially if the training lacks a philosophical or moral component [30,78]. MMA practitioners tend to exhibit higher levels of aggression compared to those practicing traditional martial arts like Oyama Karate and BJJ [43,78]. Specifically, MMA fighters show higher levels of physical and verbal aggression, while BJJ practitioners often experience a decline in aggression over time [78]. Rosario et al. [79] argues, based on reversal theory, that aggression should not be viewed as unhealthy or pathological. Instead, it is considered an essential and positive element MA&CS where fight is crucial for achieving successful performance. Mickelsson [78] even suggests that individuals with higher pre-existing aggression levels may be more drawn to disciplines like MMA. Conversely, traditional MA with a strong philosophical foundation may effectively reduce antisocial behavior [3]. Indeed, studies suggest that MA&CS training that emphasizes self-control and moral reasoning can lead to decreased aggression and bullying behavior among adolescents [68,80]. The context and type of MA&CS training can be crucial in determining its impact on aggression.

### Practical implication of the study

There are some practical implications of this study. Current research indicates that the greatest benefits from MA&CS training can be gained by young people (children and adolescents), males, with a low level of education, and those who perceive their economic status as unsatisfactory. Considering sports disciplines, coaches should take into account that novices have worse control over negative emotions than advanced people in MA&CS training. Also, athletes representing BJJ have a lower level of aggression compared to those practicing karate and MMA. If the basis for choosing the MA&CS discipline is aggression understood as a trait, coaches should work more on the externalization of control over emotions and behavior among athletes practicing karate and MMA, than BJJ or wrestling.

The role of coaches and training methods is critical in shaping the psychological landscape of athletes, affecting their emotional responses and overall well-being [81]. Coaches teach not only MA&CS techniques, but also moral code, roles, responsibilities, refereeing, and safety [82]. Ciaccioni et al. [82] developed a comprehensive educational intergenerational judo programmes, including aims, key characteristics, coaches’ roles, barriers, and facilitators. Engaging in activities and interactions across generations can lead to a positive shift in social dynamics, emphasizing the development of respect and empathy among those involved. The judo coach fulfills multiple roles, serving not only as a technical authority but also as a mentor who guides and motivates participants. Additionally, the coach acts as a role model, exemplifying positive behaviors, ensuring safe practices, and fostering strong connections [82].

### Limitations and future directions

The research has some limitations. Although the study had an adequate sample size, the individual disciplines were not equally represented due to different demographic characteristics. It could have influenced the results of this study. In the future, it would be appropriate to include more athletes of all ages and similar experiences in each MA&CS discipline. Future research should address gender differences within a more balanced sample of MA&CS athletes. In addition, cross-cultural research could answer the question, whether specific country culture contribute to aggression in particular MA&CS disciplines. Additionally, a comparative analysis of a broader range of MA&CS disciplines is warranted. The cross-sectional nature of the study also does not allow for causal conclusions. It is unclear whether individuals who are more aggressive by nature benefit more from MA&CS training, decreasing significantly with experience, or whether they drop out over the years and only those experts who were low aggressive from the beginning remain. More longitudinal studies should be conducted to answer these questions in the future.

## Conclusions

The person-centered approach identified three subpopulations and their unique characteristics related to demographic variables and aggression. The clear division between MA&CS Non-Athletes and athletes, as well ass between MA&CS Experts and MA&CS Newbies, indicates that combat training has common features among athletes and clearly differentiates them from those who do not practice combat sports. The study highlights the role of long-term training in reducing aggression levels. On the other hand, it may also be that long-term, multi-year training is only possible for those athletes who do not exhibit overly aggressive behavior. Longitudinal studies could resolve this issue in the future, taking into account the initial level of aggression at the beginning of the sports career and checking whether the more aggressive individuals dropped out in the following years or whether those with the highest level of aggression stayed and significantly reduced this level.

The study using a person-centered approach confirmed the findings of previous studies conducted using a variable-centered approach about the role of MA&CS training in reducing aggression, particularly in dimensions such as hostility, verbal aggression, and physical aggression. Current research supported the critical role of such variables as age, gender, level of education, self-reported economic status, particular MA&CS discipline, and experience in MA&CS training play in distinguishing latent class profiles. Therefore, the issue of aggression in MA&CS practitioners should be treated comprehensively as a complex phenomenon in which socio-cultural factors may be crucial. Replication studies conducted in different cultural contexts are needed. The choice of a specific MA&CS discipline may be due to some complex dimensions of individual differences that shape the sense of identity and personality as we age. Aggression related to competitive sports appears to have mechanisms different from everyday aggression, and research with athletes is crucial to understanding its unique nature. Therefore, the results of this study may be helpful in sports psychology for sports selection as well as intervention. MA&CS coaches can use the knowledge of individual differences in aggression levels between experts and novices to optimize and improve sports training in combat sports. Coaches should prioritize the development of psychological skills and well-being among young athletes, particularly focusing on males, individuals with lower levels of education, those with a low perceived economic status, and novices in MA&CS. Specifically, the present study suggests that MA&CS training should emphasize emotional and behavioral regulation for athletes in karate and mixed martial arts (MMA) more than for practitioners of Brazilian jiu-jitsu (BJJ) or wrestling.

## Supporting information

S1 File Dataset used for this study. Dataset based on data collection in Poland (2024).
(XLSX)
